# High Postoperative Serum Cortisol Level Is Associated with Increased Risk of Cognitive Dysfunction Early after Coronary Artery Bypass Graft Surgery: A Prospective Cohort Study

**DOI:** 10.1371/journal.pone.0077637

**Published:** 2013-10-15

**Authors:** Dong-Liang Mu, Li-Huan Li, Dong-Xin Wang, Nan Li, Guo-Jin Shan, Jun Li, Qin-Jun Yu, Chun-Xia Shi

**Affiliations:** 1 Department of Anesthesiology and Surgical Intensive Care Peking University First Hospital, Beijing, China; 2 Department of Anesthesiology, State Key Laboratory of Cardiovascular Disease, Fuwai Hospital, National Center for Cardiovascular Diseases, Chinese Academy of Medical Sciences and Peking Union Medical College, Beijing, China; Thomas Jefferson University, United States of America

## Abstract

**Context:**

Stress response induced by surgery is proposed to play an important role in the pathogenesis of postoperative cognitive dysfunction.

**Objective:**

To investigate the association between postoperative serum cortisol level and occurrence of cognitive dysfunction early after coronary artery bypass graft surgery.

**Design:**

Prospective cohort study.

**Setting:**

Two teaching hospitals.

**Patients:**

One hundred and sixth-six adult patients who were referred to elective coronary artery bypass graft surgery from March 2008 to December 2009.

**Intervention:**

None.

**Main Outcome Measures:**

Neuropsychological tests were completed one day before and seven days after surgery. Cognitive dysfunction was defined using the same definition as used in the ISPOCD1-study. Blood samples were obtained in the first postoperative morning for measurement of serum cortisol concentration. Multivariate Logistic regression analyses were performed to assess the relationship between serum cortisol level and occurrence of postoperative cognitive dysfunction.

**Results:**

Cognitive dysfunction occurred in 39.8% (66 of 166) of patients seven days after surgery. Multivariate Logistic regression analysis showed that high serum cortisol level was significantly associated with the occurrence of postoperative cognitive dysfunction (odds ratio [OR] 2.603, 95% confidence interval [CI] 1.371-4.944, *P* = 0.003). Other independent predictors of early postoperative cognitive dysfunction included high preoperative New York Heart Association functional class (OR 0.402, 95% CI 0.207-0.782, *P* = 0.007), poor preoperative Grooved Pegboard test score of nondominant hand (OR 1.022, 95% CI 1.003-1.040, *P* = 0.020), use of penehyclidine as premedication (OR 2.565, 95% CI 1.109-5.933, *P* = 0.028), and occurrence of complications within seven days after surgery (OR 2.677, 95% CI 1.201-5.963, *P* = 0.016).

**Conclusions:**

High serum cortisol level in the first postoperative morning was associated with increased risk of cognitive dysfunction seven days after coronary artery bypass graft surgery.

## Introduction

Cognitive dysfunction is a common central nervous system complication in patients after cardiac surgery. It refers to a subtle disorder of thought processes and may influence isolated domains of cognition such as verbal memory, visual memory, language comprehension, attention, or concentration. The diagnosis must be made according to the results of neuropsychological tests [1,2]. The reported incidences range from 50% to 70% in patients at hospital discharge and from 20% to 40% in patients six months after surgery [3].

The occurrence of postoperative cognitive dysfunction (POCD) is associated with worse outcomes including impaired daily activities (such as driving ability), less productive working status, lowered quality of life, and long-term cognitive decline [3,4]. However, the pathophysiology of POCD has not been fully elucidated [5]. Extensive clinical studies found that POCD mainly occurred after major complicated surgery (including cardiac and non-cardiac surgery) [3,4,6], but is rarely seen after minor ambulatory surgery [7]. These results suggest that the stress response induced by surgical stimuli might play an important role in the pathogenesis of POCD. 

Cortisol is one of the most important stress hormones and its secretion is proportional and positively correlated to the severity of surgical stimuli [8,9]. It has long been observed that high circulating glucocorticoids have harmful effects on human cognitive function [10]. This is because there are glucocorticoid receptors in the hippocampus and frontal lobe, the regions that are closely related with cognition. The effects of glucocorticoids on cognition follow an inverted U-shape dose response relationship; that is, cognition is impaired by sustained glucocorticoid levels that are too low or too high but is improved by proportionate glucocorticoid level [11].

In the Second International Study of Post-Operative Cognitive Dysfunction (ISPOCD2), it was found that persistent flattening in morning/afternoon ratio of salivary cortisol concentrations was significantly related to the occurrence of early POCD in patients undergoing non-cardiac surgeries [12]. In a recent study of 77 hip surgery patients, Ji and colleagues reported that plasma cortisol concentrations were negatively correlated with mini-mental state examination (MMSE) scores at 7 days postoperatively and were significantly higher in patients who developed early POCD than in those who did not [13]. We suppose that, for patients undergoing cardiac surgery, the occurrence of early POCD is also related to the elevated cortisol level after surgery. However, the relationship between circulating cortisol level and the risk of POCD has not been studied in patients after cardiac surgery. The purpose of this study was to investigate the association between postoperative serum cortisol level and occurrence of early POCD in patients undergoing CABG surgery.

## Methods

Ethical approval for this study (No. [2007]077) was provided by the Clinical Research Ethics Committee of Peking University First Hospital, Beijing, China (Chairperson Professor Xue-Jun Zhu) on 12 February 2007. This approval was accepted by the Ethics Committee of Beijing Fuwai Hospital. Written informed consents were obtained from all patients and control subjects.

### Subject enrollment

Adult patients (≥ 18 years old) who were referred to elective CABG surgery in Peking University First Hospital and Beijing Fuwai Hospital from March 2008 to December 2009 were screened. Patients were excluded if they met any of the following criteria: (1) history of cardiothoracic surgery, (2) history of schizophrenia, (3) history of adrenal gland disease, (4) history of glucocorticoid therapy for more than 7 consecutive days within 1 year (5), preoperative left ventricular ejection fraction of less than 25% (echocardiography, Simpson’s method) (6), concomitant surgery other than CABG (such as valve replacement), and (7) unable to complete neuropsychological tests before surgery. 

This prospective cohort study is continued from our previous ones [14,15]. The data of 103 patients who completed both serum cortisol measurement and perioperative neuropsychological tests during the previous studies were included in the final analyses of this paper. 

For the purpose of estimating the magnitude of practice effect for the neuropsychological tests used in this study, a group of control subjects was enrolled from friends and family members of patients with the same criteria. These individuals underwent neuropsychological testing at the same time intervals as the patients but did not undergo any surgical procedures or anesthesia. 

### Anesthesia, surgery, and postoperative care

Patients were premedicated with midazolam (7.5 mg by mouth) and morphine (10 mg intramuscularly). For some patients, penehyclidine (1.0 mg intramuscularly) was also administrated to decrease oral secretion. Anesthesia was induced with fentanyl (5 to 10 μg kg^-1^), etomidate (0.1 to 0.4 mg kg^-1^), and rocuronium (0.6 mg kg^-1^). Anesthesia was maintained with midazolam (0.05 to 0.1 mg kg^-1^), fentanyl (10 to 20 μg kg^-1^), isoflurane (0.5% to 1 % in 50% air and oxygen), and propofol (2.0 to 4.0 mg kg^-1^ h^-1^ during cardiopulmonary bypass). Supplemental doses of vencuronium were administrated to maintain muscle relaxation. Intra-operative monitoring included 5-lead electrocardiogram, radial arterial pressure, central venous pressure, pulse oxygen saturation, end-tidal expiratory carbon dioxide, nasopharyngeal temperature, bladder temperature, and urine output. A pulmonary artery catheter was inserted when necessary. 

The type of surgery (surgery with or without cardiopulmonary bypass) and the number of bypass grafts were determined by surgeons. All patients underwent CABG surgery through a median sternotomy. In patients undergoing surgery without cardiopulmonary bypass, distal anastomoses were performed with the help of an Octopus tissue stabilizer (Medtronic, Inc., Minneapolis, MN, USA). Proximal anastomoses were fashioned onto the aorta by means of a single side-clamp. Nasopharyngeal temperature was maintained above 35°C, and systolic blood pressure was kept more than 80 mm Hg throughout the procedure. For patients undergoing surgery with cardiopulmonary bypass, cardiopulmonary bypass was established with a roller pump (Stöckert Instrumente GmbH, Munich, Germany), a membrane oxygenator (Maxima Forte; Medtronic, Inc.), and a 40-μm arterial blood filter (Dideco, Miran dola, Italy). An appropriate site for cannulation and clamping was selected by surgeons after aortic palpation to avoid of atherosclerosis. Moderate hypothermia (32°C) and α-stat acid-base management were employed. Perfusion pressure was kept between 60 and 80 mmHg, and pump flow was maintained between 2 to 2.4 L min^-1^ m^-2^. After all distal anastomoses were completed, the aortic cross-clamp was removed, and proximal anastomoses were then performed by means of a single side-clamp on the aorta. 

After surgery, all patients were transferred to the intensive care unit (ICU) without extubation and were placed on mechanical ventilation. Morphine, propofol, or midazolam was administered for analgesia and sedation. Extubation and ICU discharge were decided by the attending intensivists. Hospital discharge was decided by the attending surgeon. Occurrence of postoperative complications, which were defined as medical events that required therapeutic intervention after surgery, was recorded. 

### Measurement of serum cortisol level

Blood samples were obtained in the first postoperative morning (from 7 to 8 a.m.). Serum cortisol concentration was measured with a solid-phase, competitive chemiluminescent enzyme immunoassay in a calibrated Immulite 1000 analyzer (Diagnostic Products Corporation, Los Angeles, CA, USA) as in our previous study [14]. The intra-assay and inter-assay coefficients of variation were less than 5.6% and 8.2%, respectively. The normal cortisol range is 138 to 690 nmol L^-1^ in the laboratory where measurement was performed.

### Neurocognitive assessment

Neuropsychological tests were administrated one day before surgery and seven days after surgery for both patients and control subjects in a quiet place of the general wards. The test battery was chosen according to the Statement of Consensus on Assessment of Neurobehavioral Outcomes after Cardiac Surgery [16]. It included seven tests with nine subscales and had been used in our previous studies [15,17]. Specific tests used were as follows: the Mental Control and Digit Span (forward and backward) subtests of the Wechsler Memory Scale (Chinese edition, Hunan Medical University, Hunan, China), measures of attention and concentration, with high scores indicating better function; the Visual Retention and Paired Associate Verbal Learning subtests of the Wechsler Memory Scale (Chinese edition, Hunan Medical University), measures of figural memory and verbal learning/memory, with high scores indicating better function; the Digit Symbol subtest of the Wechsler Adult Intelligence Scale-Revised (Chinese edition, Hunan Medical University), a measure of psychomotor speed, with a high score indicating better function; the Halstead-Reitan Trail Making Test (Part A), a measure of hand-eye coordination, attention, and concentration, with a low score indicating better function; and the Grooved Pegboard Test (dominant and non-dominant hand), a measure of manual dexterity, with a low score indicating better function. 

Prior to the study, two investigators (GJ Shan and J Li) were trained on psychometric test administration and relevant interview techniques by a psychiatrist. During the study phase, testing was performed and scored in a standardized manner in order to minimize inter-examiner difference. Repeat assessments for each patient were conducted by the same examiner. Parallel forms of tests were used in a randomized manner in sequential testing in order to minimize practice effect. 

Preoperative cognitive impairment was diagnosed using the definition of Hogue et al. [18] Performance on each test was compared with cognitive test data of control subjects. A low test score was defined when it was ≥ 2 SD lower than control subjects. For tests in which a higher score indicates decrement in performance, a score ≥ 2 SD higher than control subjects was considered as “low test score.” Patients were defined as having preoperative cognitive impairment if they had low test scores on two or more tests. 

POCD was diagnosed using the same definition as used in the ISPOCD1-study and our previous studies [6,15,17]. To quantify practice effect, baseline scores were compared with subsequent test results seven days later in control subjects. For patients, preoperative scores were compared with postoperative test results, subtracted the average practice effect from these changes, and then divided the result by the control-subject SD to obtain a Z score for each test. The test results were adjusted so that a positive Z score indicated deterioration from the baseline test. The Z scores of all tests in an individual patient were then summarized and divided by the SD for this sum of Z scores in the control subjects, creating a combined Z score. A patient was defined as having POCD when two Z scores in individual tests or the combined Z score were 1.96 or more. 

### Sample size calculation and statistical analysis

The primary objective of the study was to test the null hypothesis that the serum cortisol concentration on the first postoperative morning is identical in two groups of patients: those who developed cognitive dysfunction seven days after surgery and those who did not. According to our previous studies, we assumed that POCD occurred in 50% of patients and that the serum cortisol concentration would be 470 ± 300 nmol L^-1^ in the non-POCD patients and 620 ± 280 nmol L^-1^ in the POCD patients [14,15]. The calculated sample size that would provide 90% power to see this difference based on a two-tailed significance level of 0.05 is 160 patients. Considering an estimated attrition rate of 10%, the final sample size was increased to a total of 176 patients. The sample size calculation was performed with STATA 10.0 software (StataCorp LP, College Station, Texas). 

Continuous variables are presented as mean ± SD or median (inter-quartile range). Data were compared with the use of independent samples t test or Mann-Whitney U test. Categorical variables are presented as number of patients (percentage). Data were compared with the use of chi-square test or Fisher’s exact test. The relationship between serum cortisol level and the occurrence of early POCD was assessed with multivariate logistic regression analysis. First, baseline and perioperative variables were evaluated for univariate association with POCD. Variables that were significant in univariate analyses (*P* ≤ 0.05) were included in a multivariate Logistic regression model to determine the risk-adjusted predictors of POCD. Two-sided *P* values of less than 0.05 were regarded as significant. All statistical analyses were performed with the SPSS statistical package version 14.0 (SPSS Inc, Chicago, Ill). 

## Results

**Figure 1:**
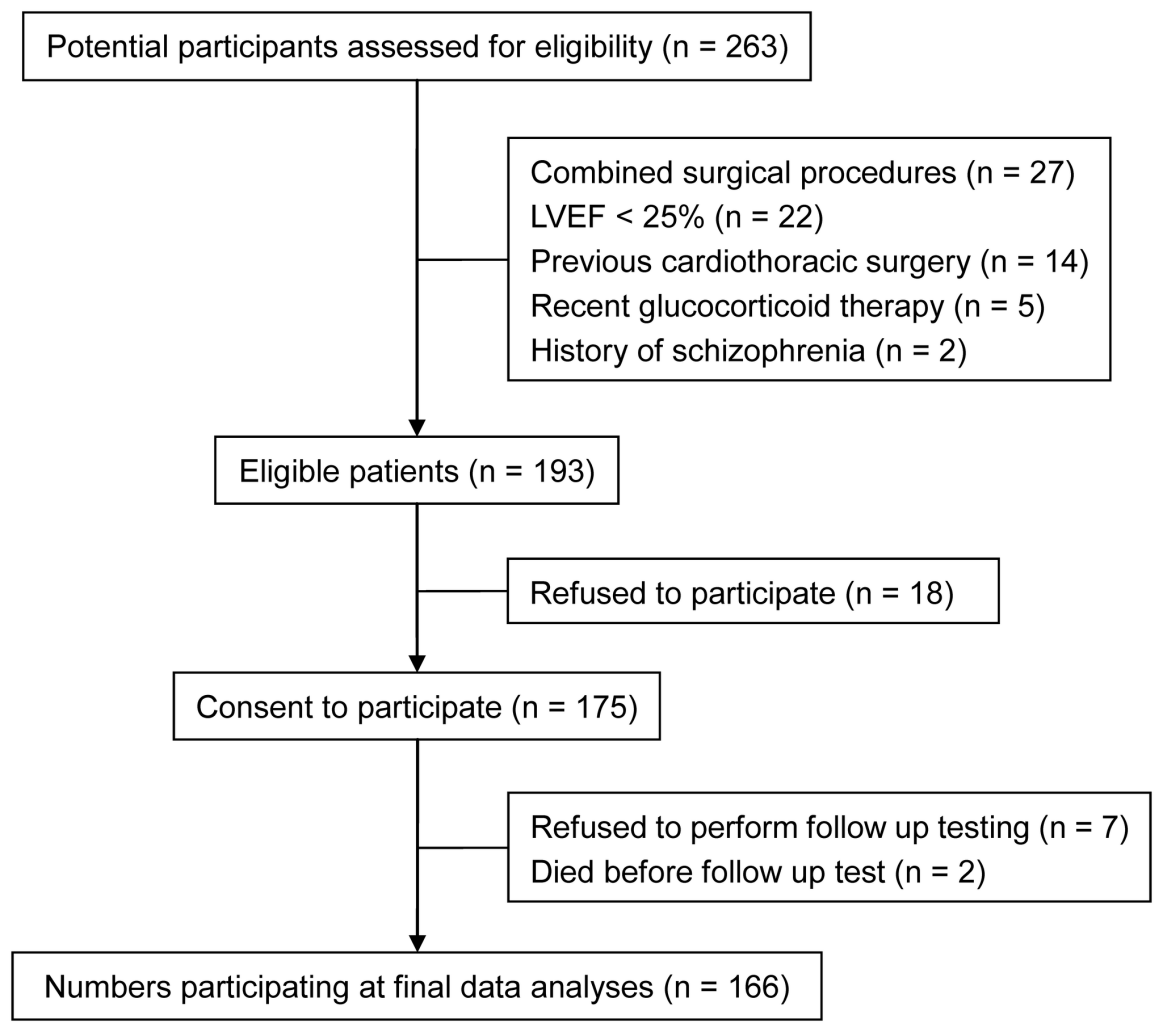
Flow chart of the study. LVEF = left ventricular ejection fraction.

During the study period, 263 patients were screened and 193 of them matched the criteria of selection. Among the eligible patients, 175 gave written informed consents and were enrolled in the study. During the postoperative period, two patients died during hospitalization, resulting in an overall in-hospital mortality rate of 1.1% (2 of 175). These two patients died of heart failure and intractable ventricular fibrillation on the second and the third postoperative day, respectively. Seven patients refused to perform postoperative neuropsychological testing. At last, 166 patients were included in final data analyses (Figure 1). The baseline characteristics and perioperative variables are listed in Tables 1 and 2.

**Table 1:** Baseline characteristics.

**Variables**	**All patients (n=166)**	**Non-POCD patients (n=100)**	**POCD patients (n=66)**	***P* value**
Age (year)	60.0 ± 8.9	59.2 ± 9.4	61.2 ± 8.1	0.148
BMI (kg m^-2^)	26.0 ± 2.9	26.1 ± 3.0	25.9 ± 2.9	0.696
Education (year)	10.9 ± 3.4	10.7 ± 3.2	11.3 ± 3.6	0.291
Female gender	25 (15.1%)	15 (15.0%)	10 (15.2%)	0.979
Previous history				
Hypertension	99 (59.6%)	52 (52.0%)	47 (71.2%)	0.014
Myocardial infarction	69 (41.6%)	45 (45.0%)	24 (36.4%)	0.269
Diabetes mellitus	58 (34.9%)	27 (27.0%)	31 (47.0%)	0.008
Hyperlipidemia	62 (37.3%)	37 (37.0%)	25 (37.9%)	0.909
Arrhythmia^a^	26 (15.7%)	16 (16.0%)	10 (15.2%)	0.883
Stroke	21 (12.7%)	11 (11.0%)	10 (15.2%)	0.431
COPD	7 (4.2%)	2 (2.0%)	5 (7.6%)	0.175
Chronic Smoking	85 (51.2%)	48 (48.0%)	37 (56.1%)	0.309
Preoperative LVEF (%)^b^	58.9 ± 10.7	57.8 ± 10.8	60.7 ± 10.3	0.086
CCS classification				0.975
I	29 (17.5%)	18 (18.0%)	11 (16.7%)	
II	100 (60.2%)	60 (60.0%)	40 (60.6%)	
III	33 (19.9%)	20 (20.0%)	13 (19.7%)	
IV	4 (2.4%)	2 (2.0%)	2 (3.0%)	
NYHA classification				0.013
I	33 (19.9%)	14 (14.0%)	19 (28.8%)	
II	111 (66.9%)	68 (68.0%)	43 (65.2%)	
III	22 (13.3%)	18 (18.0%)	4 (6.1%)	
Preoperative EuroSCORE	2.0 (1.0-4.0)	2.0 (1.0-3.0)	2.0 (1.0-4.3)	0.443

Data was presented as mean ± SD, number (percentage) or median (inter-quartile range).

^a^ Indicated those necessitating medical treatment.

^b^ Results of echocardiography (Simpson’s method).

POCD = postoperative cognitive dysfunction; BMI = body mass index; COPD = chronic obstructive pulmonary disease; LVEF = left ventricular ejection fraction; CCS = Canadian Cardiac society; NYHA = New York Heart Association; EuroSCORE = European system for cardiac operative risk evaluation.

**Table 2:** Perioperative variables.

**Variables**	**All patients (n=166)**	**Non-POCD patients (n=100)**	**POCD patients (n=66)**	***P* value**
Duration of anesthesia (hour)	4.87 ± 1.05	4.83 ± 1.01	4.92 ± 1.13	0.572
Dose of etomidate during induction (mg/kg)	0.19 ± 0.11	0.18 ± 0.11	0.21 ± 0.09	0.207
Use of anticholinergics during anesthesia				
Penehyclidine^a^	41 (24.7%)	17 (17.0%)	24 (36.4%)	0.005
Atropine^b^	21 (12.7%)	14 (14.0%)	7 (10.6%)	0.520
Use of corticosteroids during anesthesia^c^	64 (38.6%)	43 (43.0%)	21 (31.8%)	0.147
Duration of surgery (hour)	3.66 ± 1.106	3.55 ± 1.05	3.82 ± 1.06	0.103
Surgery under cardiopulmonary bypass	58 (34.9%)	30 (30.0%)	28 (42.4%)	0.100
Blood transfusion of at least 400 mL	36 (21.7%)	22 (22.0%)	14 (21.2%)	0.904
Number of bypass grafts	3.0 ± 1.0	2.9 ± 1.0	3.1 ± 1.1	0.231
Duration of sedation in ICU (hour)	8.8 (6.0-11.4)	7.8 (5.0-10.0)	9.8 (7.0-12.0)	0.008
Duration of mechanical ventilation in ICU (hour)	14.8 (11.5-18.0)	14.5 (11.0-18.9)	14.9 (12.2-18.0)	0.612
Duration of ICU stay (hour)	42.5 (22.0-70.0)	43.0 (23.0-71.5)	29.2 (21.0-68.5)	0.056
Serum cortisol concentration (nmol L^-1^)^d^	517.9 ± 276.0	467.0 ± 260.8	597.4 ± 282.4	0.003
Serum cortisol level^d^				< 0.001
Level 1	17 (10.4%)	13 (13.0%)	4 (6.3%)	
Level 2	103 (62.8%)	71 (71.0%)	32 (50.0%)	
Level 3	44 (26.8%)	16 (16.0%)	28 (43.8%)	
Postoperative complications^e^				
Cardiac insufficiency	22 (13.3%)	11 (11.0%)	11 (16.7%)	0.292
Arrhythmia	4 (2.4%)	2 (2.0%)	2 (3.0%)	0.650
Myocardial infarction	2 (1.2%)	0 (0.0%)	2 (3.0%)	0.157
Pleural effusion	11 (6.6%)	2 (2.0%)	9 (13.6%)	0.007
Respiratory insufficiency	5 (3.0%)	1 (1.0%)	4 (6.1%)	0.082
Pneumonia	1 (0.6%)	0 (0.0%)	1 (1.5%)	0.398
Pulmonary atelectasis	1 (0.6%)	1 (1.0%)	0 (0.0%)	1.000
Surgical bleeding	1 (0.6%)	0 (0.0%)	1 (1.5%)	0.398
Sepsis	6 (3.6%)	3 (3.0%)	3 (4.5%)	0.683
Occurrence of postoperative complications^e^	48 (28.9%)	20 (20.0%)	28 (42.4%)	0.002
Duration of postoperative hospital stay (day)	8.0 (7.0-14.0)	8.0 (7.0-12.0)	10.5 (7.0-14.0)	0.095

Data was presented as mean ± SD, number (percentage), or median (inter-quartile range).

^a^ Used as premedication (1.0 mg IM).

^b^ Used as anti-bradycardia agent (0.3-1.0 mg IV).

^c^ Mainly dexamethasone (10 mg) for prophylaxis of postoperative nausea and vomiting.

^d^ The normal range is 138 to 690 nmol L^-1^. Level 1 indicates a serum cortisol concentration of less than 138 nmol L^-1^, level 2 between 138 and 690 nmol L^-1^, and level 3 greater than 690 nmol L^-1^. Excluded two patients with missing data.

^e^ Indicated those that occurred within seven days after surgery.

POCD = postoperative cognitive dysfunction; ICU = intensive care unit.

During the same period, 59 control subjects were enrolled for repeated neuropsychological testing. The control subjects and patients are comparable in age (61.8 ± 7.5 years vs. 60.0 ± 8.9 years, P = 0.176), female gender ratio (13 of 59 [22.0%] versus 25 of 166 [15.1%], P = 0.219), and education (11.5 ± 3.5 years vs. 10.9 ± 3.4 years, P = 0.272). The baseline neuropsychological test results of control subjects and patients are listed in Table 3.

**Table 3:** Baseline neuropsychological test results.

**Variables**	**Control subjects (n = 59)**	**All patients (n=166)**	**Non-POCD patients (n=100)**	**POCD patients (n=66)**	***P* value**
Mental Control	93.6 ± 10.0	90.3 ± 15.8	90.1 ± 15.2	90.5 ± 16.7	0.888
Visual Retention	11.4 ± 2.3	11.0 ± 2.8	10.8 ± 2.9	11.3 ± 2.6	0.260
Paired Associate Verbal Learning	17.1 ± 2.7	15.5 ± 3.1	15.6 ± 3.0	15.5 ± 3.2	0.769
Digit Span Forward	8.2 ± 1.1	7.8 ± 1.4	7.6 ± 1.5	8.0 ± 1.3	0.053
Digit Span Backward	4.8 ± 1.3	4.7 ± 3.0	4.4 ± 1.2	5.1 ± 4.5	0.243
Digit Symbol	37.8 ± 10.9	33.6 ± 21.1	32.3 ± 10.2	35.5 ± 31.2	0.341
Trail Making Part A	113.7 ± 38.2	117.7 ± 44.6	120.9 ± 46.6	112.8 ± 41.4	0.253
Grooved Pegboard Dominant-hand	77.8 ± 16.6	73.7 ± 19.8	72.5 ± 17.5	75.5 ± 22.9	0.341
Grooved Pegboard Nondominant-hand	83.1 ± 22.6	75.7 ± 22.9	72.0 ± 23.0	81.4 ± 21.7	0.009
Preoperative cognitive impairment	3 (5.1%)	18 (10.8%)	10 (10.0%)	8 (12.1%)	0.667

Data was presented as mean ± SD or number (percentage).

Cognitive dysfunction occurred in 66 patients seven days after surgery (39.8% [66/166]) and in four control subjects at second test session (6.8% [4/59]) (*P* < 0.001). Serum cortisol concentration was significantly higher in patients who developed early POCD than in those who did not (*P* = 0.003, Table 2). 

**Figure 2:**
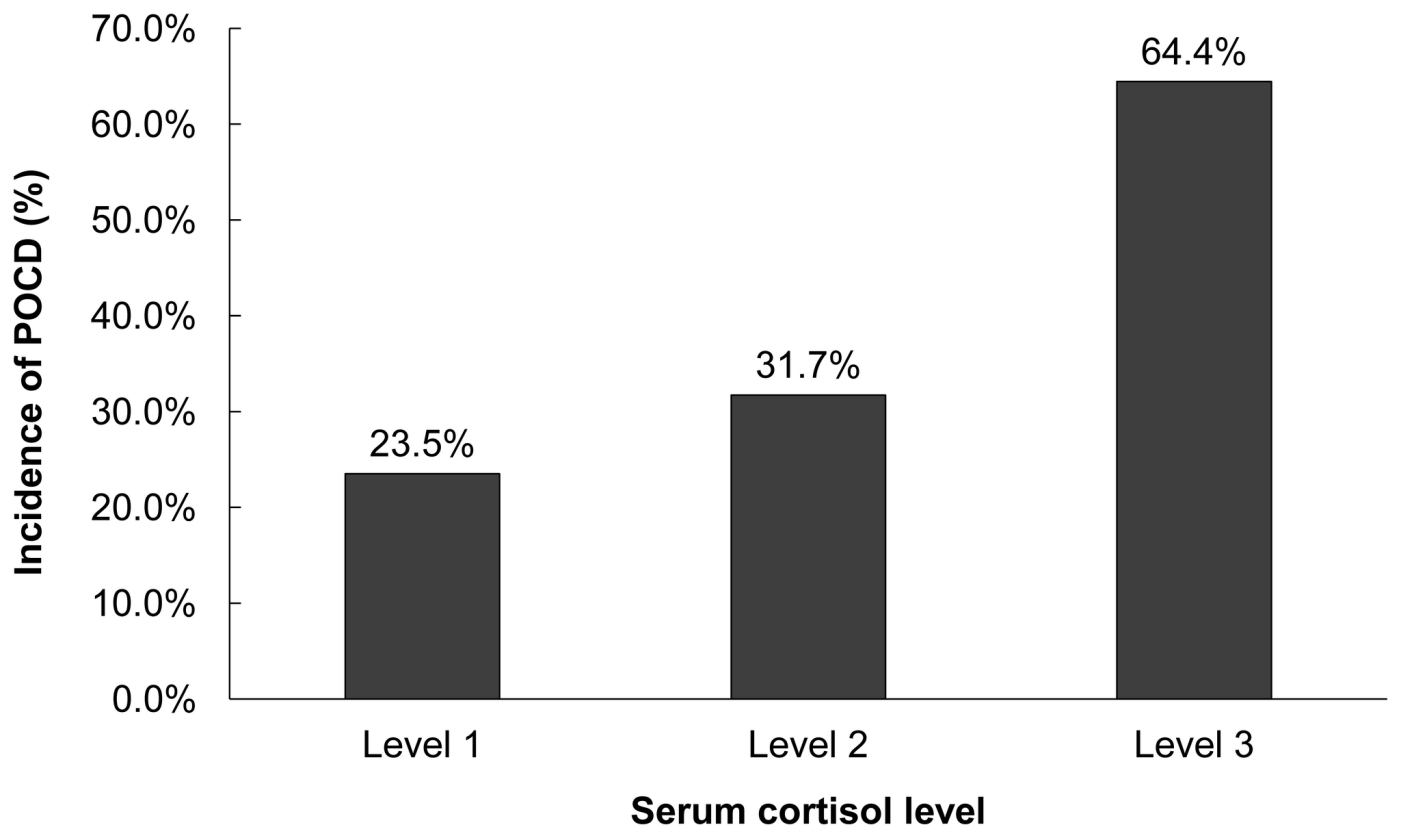
Relationship between the serum cortisol level and the incidence of cognitive dysfunction seven days after coronary artery bypass graft surgery. Patients with an elevated serum cortisol level had a significantly higher incidence of postoperative cognitive dysfunction (*P* < 0.001). The normal range of serum cortisol concentration is 138 to 690 nmol L^-1^. Level 1 indicates a serum cortisol concentration of less than 138 nmol L^-1^, level 2 between 138 and 690 nmol L^-1^, and level 3 of greater than 690 nmol L^-1^.

According to the normal range of serum cortisol concentration in the laboratory where measurement was performed, we divided cortisol concentration into three levels, i.e., level 1 (< 138 nmol L^-1^), level 2 (138-690 nmol L^-1^), and level 3 (> 690 nmol L^-1^)^13^. Patients with high serum cortisol level had a significantly higher incidence of POCD (*P* < 0.001, Figure 2). 

Variables that were associated with the occurrence of early POCD are listed in Table 4. After correction of confounding factors, high serum cortisol level in the first postoperative morning remained as an independent predictor of the occurrence of cognitive dysfunction seven days after surgery (odds ratio [OR] 2.603, 95% confidence interval [CI] 1.371 to 4.944, *P* = 0.003). Other independent predictors of early POCD included high preoperative New York Heart Association functional class, poor preoperative Grooved Pegboard test score of nondominant hand, use of penehyclidine as premedication, and occurrence of complications within seven days after surgery. 

**Table 4:** Predictors of postoperative cognitive dysfunction.

	**Univariate analyses^a^**		**Multivariate logistic regression analysis^b^**	
	***P* value**		**Odds ratio (95% confidence interval)**	***P* value**
History of hypertension	0.014		-	-
History of diabetes mellitus	0.009		-	-
Preoperative NYHA functional classification (class)	0.004		0.402 (0.207-0.782)	0.007
Preoperative Grooved Pegboard Nondominant-hand (second)	0.012		1.022 (1.003-1.040)	0.020
Use of penehyclidine as premedication	0.005		2.565 (1.109-5.933)	0.028
Serum cortisol level (level)^c^	< 0.001		2.603 (1.371-4.944)	0.003
Occurrence of postoperative complications^d^	0.002		2.677 (1.201-5.963)	0.016

^a^ Occurrence of postoperative cognitive dysfunction was modelled as a function of a single predictor.

^b^ Occurrence of postoperative cognitive dysfunction was modelled as a function of all predictors that were significant (*P* ≤ 0.05) in the univariate analyses. Multivariate logistic regression analysis was performed by using a Forward (Conditional) stepwise procedure.

^c^ The normal range is 138 to 690 nmol L^-1^. Level 1 indicates a serum cortisol concentration of less than 138 nmol L^-1^, level 2 between 138 and 690 nmol L^-1^, and level 3 of greater than 690 nmol L^-1^. Excluded two patients with missing data.

^d^ Indicated those that occurred within seven days after surgery.

NYHA = New York Heart Association.

Replacement of serum cortisol level with serum cortisol concentration in nmol L^-1^ did not change the results, high serum cortisol concentration was also an independent predictor of early POCD (OR 1.002, 95% CI 1.000-1.003, *P* = 0.017). 

## Discussion

In the present study, we found that high serum cortisol level in the first postoperative morning was associated with increased risk of cognitive dysfunction seven days after surgery. Furthermore, poor preoperative Grooved Pegboard test score of nondominant hand, use of penehyclidine as premedication, and occurrence of complications within seven days after surgery was also associated with increased risk of early POCD. Poor preoperative cardiac function (as assessed by NYHA classification) was associated with decreased risk of early POCD. 

There is marked variability in the measurement and definition of POCD [19]. In this study, the neuropsychological tests as suggested by the Statement of Consensus on Assessment of Neurobehavioral Outcomes after Cardiac Surgery were used, and the definition of POCD as used in the ISPOCD1-study was adopted, so that our results could be compared with others [6,16]. We found again that the incidence of POCD was high early after CABG surgery although it was slightly lower than our previous results [15,17]. However, it was well within the range of recently reported incidences in similar patients populations [20]. 

The causative factors of early POCD are considered to be multifactorial [5,20]. Predisposing factors (patient-related factors) may include age, pre-existing neurodegeneration and other comorbidities [21,22]. Precipitating factors may include impaired cerebral perfusion and oxygenation during surgery, inflammatory response provoked by surgical stress, and postoperative sleep deprivation [7,23-27]. When discussing these factors, we must note that patient-related factors, although may be helpful to identify high risk patients, are usually unmodifiable. Whereas by changing perioperative management of patients, such as minimizing the severity of surgical injury, it might be possible to decrease the incidence of POCD. However, the relationship between the severity of surgical stress and the occurrence of POCD has not been clearly demonstrated. 

Cortisol is frequently used as a marker of surgical stress because its secretion is proportional and positively correlated to the severity of surgical stimuli [8,9]. There is a reciprocal control mechanism between the brain and the release of glucocorticoid hormones, i.e., the hypothalamic-pituitary-adrenal (HPA) axis. Under stressful conditions, the brain promotes cortisol secretion via hypothalamic corticotrophin releasing hormone. On the other hand, glucocorticoids produce negative feedback mechanisms by action at specific receptors in the hypothalamus. Furthermore, the hippocampus also plays an important role in inhibiting the HPA stress response, at least partially by activation of glucocorticoid receptors in this region [28]. 

Glucocorticoid receptors in the hippocampus are gradually lost in the process of normal aging. This results in attenuation of the negative feedback mechanism that inhibits further secretion of corticosteroids [11,29]. Numerous studies indicate that there is a strong correlation between elevated cortisol level and hippocampal damage and/or glucocorticoid receptor loss, both of which are associated with learning impairments [10]. Loss of glucocorticoid receptors and attenuation of the negative feedback mechanism make the elderly more prone to develop sustained hypersecretion of glucocorticoids and, thus, cognitive complications after major surgery. Our results also showed that there was a significant correlation between age and postoperative serum cortisol concentration (Pearson correlation coefficient = 0.195, *P* = 0.012). In patients after non-cardiac surgery, it was found that circadian rhythm disturbance of cortisol level (i.e., persistent flattening in am/pm ratio) was significantly related to the occurrence of early POCD [12]. However, the relationship between serum cortisol level and the occurrence of POCD is unknown in patients undergoing cardiac surgery. 

We did not monitor the time-course changes of serum cortisol level in this study. Blood samples were collected in the morning of the first postoperative day. This time-point was selected in order to get a relatively high serum cortisol level. In the study of Rasmussen et al. [12], peak salivary cortisol levels were found in the morning of the first postoperative day. For patients who underwent cardiac surgery, serum cortisol concentrations peaked on the first postoperative day or from 4 to 12 hours after surgery [30,31]. In another study, it was found that serum cortisol level on the first postoperative day was significantly associated with the occurrence of postoperative inflammatory complication after cardiac surgery [32]. Our study demonstrated that high serum cortisol level in the first postoperative morning was significantly associated with increased risk of cognitive dysfunction seven days after CABG surgery. In the present study, we also found that 10.4% of patients (17 of 164) had serum cortisol concentration lower than normal, perhaps because of the use of etomidate during anesthesia induction. But this did not seem to produce significant adverse effects since none of these patients developed circulatory collapse and received glucocorticoid replacement therapy during hospitalization. And there was no significant correlation between etomidate dose and serum cortisol concentration (Pearson correlation coefficient = 0.035, *P* = 0.651). 

However, whether there is a causal relationship between the elevated cortisol level after surgery and the occurrence of early POCD remains unknown. Studies found that chronic glucocorticoid therapy produced memory deficit in patients [33]. And inhibiting the effects of glucocorticoids (by inhibiting the effects of 11β-hydroxysteroid dehydrogenase) helped to preserve cognitive function in high risk patients [34]. It is possible that higher and more prolonged cortisol response to surgical stress in the elderly plays an important role in the pathogenesis of POCD. On the other hand, there are also studies showing beneficial effects of glucocorticoid therapy on cognitive function. Cardiac surgery often induces significant inflammatory response which may be the underlying cause of POCD [35,36]. Glucocorticoids have been used as anti-inflammatory agents to reduce postoperative complications [37]. In a recent large, multicenter clinical trial of patients undergoing cardiac surgery, Dieleman et al. [38] reported that, although intraoperative high-dose dexamethasone (1mg/kg) did not reduce the 30-day incidence of major adverse events, it decreased the incidence of postoperative delirium compared with placebo (9.2% vs. 11.7%, RR 0.79, *P* = 0.006). And it is well known that delirium is closely related to the occurrence of POCD [15]. Therefore, it is also possible that the rising serum cortisol is actually a neuroprotective factor. The inflammatory response to surgical stimuli, rather than the cortisol response, is the real cause of POCD. An important reason that led to the above conflicting results is the duration of glucocorticoid therapy. A single dose of dexamethasone was administered in the study of Dieleman et al. [38], whereas chronic glucocorticoid therapy usually means a much longer duration. It is necessary to further clarify the relationship between the duration of high cortisol level after surgery and the occurrence of POCD. 

It is difficult to understand why poor preoperative cardiac function was associated with decreased risk of POCD in our study. Cardiac dysfunction is associated with cognitive impairment in patients with heart disease [39,40]. In the present study, there was also a weak but significant correlation between baseline NYHA class and preoperative cognitive impairment (Spearman correlation coefficient = 0.175, *P* = 0.024). CABG surgery can significantly improve cardiac function, especially in patients with impaired preoperative left ventricular function [41-43]. In cardiac transplantation candidates, it was found that cognitive function was significantly improved after transplantation [44]. It is possible that, for patients with high preoperative NYHA class, CABG surgery improved cognitive function (thus decreased the risk of POCD) by improving cardiac function. However, this requires further study. 

Preoperative cognitive impairment is thought to be an important risk factor of POCD [45]. However, data on this subject is equivocal [46]. In the present study, preoperative cognitive impairment existed in 10.8% of patients. This is lower than the previously reported incidences in patients scheduled for cardiac surgery (from 25% to 45%) [18,47]. Possible reasons included: (1) patients enrolled in this study were younger than in previous ones; (2) patients who were unable to complete preoperative neuropsychological tests were excluded from the present study. Our study found that poor preoperative manual dexterity of non-dominant hand, but not overall preoperative cognitive impairment, predicted the occurrence of early POCD. 

Chronic use of medications with anticholinergic activity has significant negative effects on cognitive function of older adults [48]. However, the relationship between perioperative use of anticholinergic drugs and the occurrence of POCD has not been clearly demonstrated. Penehyclidine hydrochloride is a new anticholinergic medicine with greater selectivity for M1 and M3 muscarinic receptors [49]. Since it has less M2 receptor-associated cardiovascular side effects, some Chinese anaesthesiologists prefer to use it as a premedication to decrease salivary secretion [50]. Our results showed that, for patients undergoing CABG surgery, perioperative use of penehyclidine significantly increased the risk of early POCD. 

Occurrence of postoperative complications usually means a more eventful postoperative recovery. In the present study, complications that occurred within seven days after surgery (i.e., before postoperative neuropsychological test session) were recorded. Our study also found that the occurrence of postoperative complications was significantly associated with increased risk of early POCD. 

Most, if not all, studies of POCD report that increased age is a strong predictive factor of POCD [51]. Some studies also find that lower educational level is associated with increased risk of POCD [51,52]. However, we did not find these associations in the present study. One possible reason was that our sample size was relatively small. Another possible reason was that the ranges of age and educational duration were relatively narrow in patients of this study. These made the effects of age and educational level difficult to detect. 

There are several limitations of this study. First, we did not measure baseline serum cortisol concentration. Therefore, we were unable to determine if patients with elevated baseline serum cortisol level were more prone to develop POCD. In older adults, high baseline cortisol level was associated cognitive impairment [10]. Second, serum cortisol concentrations were measured at only one time-point after surgery. The time-course changes were not monitored. We were unable to determine if prolonged duration of high serum cortisol level was also correlated with the occurrence of POCD. In medical patients, chronic glucocorticoid therapy produced cognitive decline [33]. Third, our study did not reveal the causal relationship between the elevated cortisol level and the occurrence of early POCD. High cortisol level may have a direct impact on the cognitive function, but it is also possible that high cortisol level merely reflects the stress response to perioperative stimuli. Therefore, further study is needed to illuminate the mechanisms by which circulating cortisol level may affect cognitive function after surgery. 

In conclusion, our study found that high serum cortisol level in the first postoperative morning was associated with increased risk of cognitive dysfunction seven days after CABG surgery. 
